# Correlations between Physical Activity Participation and the Environment in Children and Adolescents: A Systematic Review and Meta-Analysis Using Ecological Frameworks

**DOI:** 10.3390/ijerph18179080

**Published:** 2021-08-28

**Authors:** Longxi Li, Michelle E. Moosbrugger

**Affiliations:** Department of Physical Education and Health Education, Springfield College, Springfield, MA 01109, USA; lli@springfieldcollege.edu

**Keywords:** children, adolescents, ecological frameworks, physical activity, health, comprehensive meta-analysis

## Abstract

Physical activity (PA) and sports are efficient ways to promote the young generation’s physical and mental health and development. This study expected to demonstrate the complexity of correlates associated with children’s and adolescents’ non-organized PA participation. Following the Preferred Reporting Items for Systematic Review and Meta-analysis Protocols (PRISMA), a systematic review and meta-analysis were applied. Seven electronic databases were systematically searched to identify eligible articles based on a series of inclusion and exclusion criteria. The internal validity of the systematic reviews thus identified was evaluated using a validated quality instrument. Calculations were produced in SPSS 27.0 and Comprehensive Meta-Analysis 3.3. Thirty-nine eligible studies (*N* = 324,953) with moderate to high quality were included. No potential publication bias was detected using statistical analyses. The meta-analysis revealed that the overall ecological factors correlated positively with children and adolescents’ PA; the meta-analytic average of the correlations was (*′r* = 0.32, *p* < 0.001). Results from subgroup analysis indicated that theory-based influence factors achieved moderate effect with boys (*′r* = 0.37, *p* < 0.001) and girls (*′r* = 0.32, *p* < 0.001) in PA participation. Interestingly, higher correlations were found between ecological factors and twins’ PA participation (*′r* = 0.61, *p* = 0.001). Further, individual (*′r* = 0.32, *p* < 0.001), macro-, and chronosystems factors (*′r* = 0.50, *p* < 0.001) appeared slightly more influential than microsystems factors (*′r* = 0.28, *p* < 0.001) on children and adolescents’ PA participation. Although findings from the included studies covered were to some extent heterogeneous, it is possible to identify consistent correlates of PA in children and adolescents. The results supported that PA is a complex and multi-dimensional behavior, which is determined by numerous biological, psychological, sociocultural, and environmental factors. Future studies that focus on the integration effect of macrosystem and chronosystem environmental factors, and apply longitudinal designs and objective measurements are encouraged to further unfold the complexity of the ecological system and its implications in promoting children and adolescents’ PA participation.

## 1. Introduction

Physical activity (PA) plays a critical role in children and adolescents’ lifelong development [1,2,3,4]. However, participation in PA decreases with age [5,6,7]; the decline is greater in girls than boys [8], and computers and social media have decreased the need and desire for children to move and play [9]. Nowadays, the issue of physical inactivity is even more concerning due to the influence of remote learning settings under the global pandemic [10]. The challenges associated with getting kids active every day should be met with age-appropriate physical activities, enthusiastic leadership, and support from family and community [11]. Hence, synthesizing previous empirical evidence and examining what type of factors can effectively influence daily active play and PA behaviors in childhood and adolescence is a critical and urgent task.

PA participation shows positive impacts on an individual’s psychological and physiological wellbeing, especially in adolescent development [12,13,14,15,16]. Childhood and adolescence represent important developmental periods where lifelong habits can be established to promote healthy lifestyles and reduce the risk of chronic disease in early adulthood [3]. It is critical to target health behaviors as a method of primary prevention early in the developmental course because children and adolescents who do not participate in adequate PA, have a poor diet, and begin smoking are at increased risk for continuing these patterns and experiencing poor health outcomes as adults [17,18]. Although previous studies have addressed the effects of health intervention programs in children and adolescents, no systematic review and meta-analysis exists that has examined PA and the surrounding environments using ecological frameworks.

Ecological frameworks have been common in health promotion studies in the last 20 years [19]. The frameworks were supported by evidence that multiple systems influence lifelong motor development and wellbeing, thus linking the outcomes back to the systems in order to further explore the synthesis of the system impacts on adolescent PA participation, health status, and motor development [13]. As the footstone to its bifurcation models, the Bronfenbrenner ecological framework [20] is primarily focused on the interactive and cumulative effect on change regarding interaction connecting individuals and their contextual surroundings manifested as mental and PA, which is the primary focus in this study [21,22].

McLeroy and colleagues [23] proposed the socioecological model (SEM) that emphasized the role of both social and environmental factors reflecting Bronfenbrenner’s four levels. SEM demonstrated that health was affected by the interaction between the characteristics of the individual, the community, and the environment that includes the physical, social, and political components, which provided a theoretical framework to examine multiple factors influencing PA behavior [24,25]. Specifically, SEM examines interactive relationships between the individual and multiple levels of the environment to understand when and how people initiate, adopt, and maintain physically active lifestyles [19]. Over time, the systems approaches (SA) and youth physical activity promotion (YPAP) model added specificity to SEM, focusing on the correlates of PA behavior change in youth [25,26]. The basis of the models was the assumption that the combination of individual, social environmental, and physical environmental factors would best explain PA. Both SA and YPAP models applied a social ecological approach, which was rooted in Bronfenbrenner’s [20] ecological framework [25,27]. Although the SA and YPAP models provided an attractive conceptual basis for the ecological framework, there is limited theoretical and practical evidence of those models in examining PA participation in children and adolescents [28,29]. 

This study provides a perspective to review the previous literature and influence factors associated with ecological frameworks in the last two decades. Based on the protocol established by Li and colleagues [21], correlations were basically categorized into individual, micro (family, peers, neighborhood, and school), exo (extended family), macro (country-level physical environments, policy, and sociocultural context), and chrono (considering the timeline of change). This study aimed to broaden the scope of the knowledge base by examining variability in effect sizes using study-level variables and then to explore the relative impact of influence factors within ecological systems.

## 2. Method

Bibliographic databases served as the primary method of identifying eligible studies. Manual searches of relevant papers were also carried out. The current review also conducted backward reference searches of other reviews and consulted with experts in the field. Since this study analyzed previous findings, ethics approval was exempted. This review protocol was registered in the PROSPERO international prospective register of systematic reviews, the registration number: CRD42021244918.

### 2.1. Search Strategy

The search strategy included use of the following databases: PubMed, SPORTDiscus, PsycInfo, MEDLINE Complete, ERIC, Dimensions, and Academic Search Complete. The search strategy was built on the systematic review protocol established by Li and colleagues [21]. Keyword combinations employed in the search are illustrated at Table 1. Beyond the database search, two additional references were extracted from the reference list of the following 10 review articles [13,26,30,31,32,33,34,35,36,37]. Two additional references were found within full-text articles being screened for inclusion [38,39]. 

### 2.2. Inclusion and Exclusion Criteria

#### 2.2.1. Study Designs

The inclusion criteria for studies were as follows: published in English during the last 20 years; original empirical studies including randomized controlled trials (RCTs), observational studies (non-randomized studies, or NRSs); moreover, quantitative data from mixed-method studies were included. Studies were excluded if classified as a systematic review or meta-analysis.

#### 2.2.2. Participants

The inclusion criteria for studies were as follows: dealt with children and adolescents aged between 3 and 18 years at the start of the study [3,40]. Studies were excluded if participants were older than 18 years old at the start of the study; or adolescents and children who were affected by a particular disease, disorder, injury, or trauma at the time of the intervention were targeted.

#### 2.2.3. Intervention

All types of influential factors associated with Bronfenbrenner’s ecological framework (e.g., individual, micro-, exo-, macro-, chrono-system factors) were accepted regarding the inclusion criteria. Studies had to include participants who were exposed within at least one of the ecological factors that resulted in PA participation. School physical education (PE) sessions and sports meetings were considered as one of the influential factors in schools that was categorized as microsystem.

#### 2.2.4. Outcome Measures

In this study, all quantitative measurement results of PA within the selected studies were considered as outcomes. The effects of ecological factors on participants’ PA were measured by intensity, duration (e.g., per day/week), and frequency. Specific to intensity, the selected studies had as outcomes any measure of overall or general PA, light PA, vigorous PA (VPA), moderate PA, and moderate to vigorous PA (MVPA). Considering self-reported PA was correlated with accelerometer data and these correlations tended to be moderate to high, both objectively measured and self-reported PA participation data were accepted [30,41]. Secondary outcomes mainly involved adverse events, parent-reported, and self-reported outcomes. Varying continuous PA data were included in alignment with the nature of correlational design and added heterogeneities to strengthen the ecological validity of the results [42]. Study measurements and PA correlations are exhibited in Supplementary Materials File A. 

### 2.3. Studies Selection and Data Extraction

**Figure 1:**
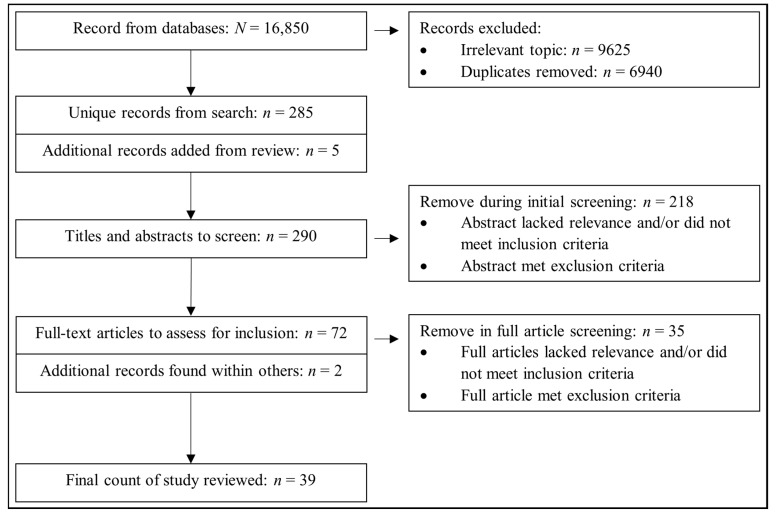
PRISMA flowchart of searching and screening.

Titles and abstracts were screened independently by two authors. Mendeley software was used for recording and managing related literature. Second, screening full texts of the selected studies, the authors checked suitable studies against the inclusion criteria. Finally, the authors generated studies together and then selected studies were determined. The selection process of suitable literature is illustrated in the Preferred Reporting Items for Systematic Review and Meta-analysis (PRISMA) flowchart (Figure 1).

### 2.4. Assessment of Risk of Bias

**Figure 2:**
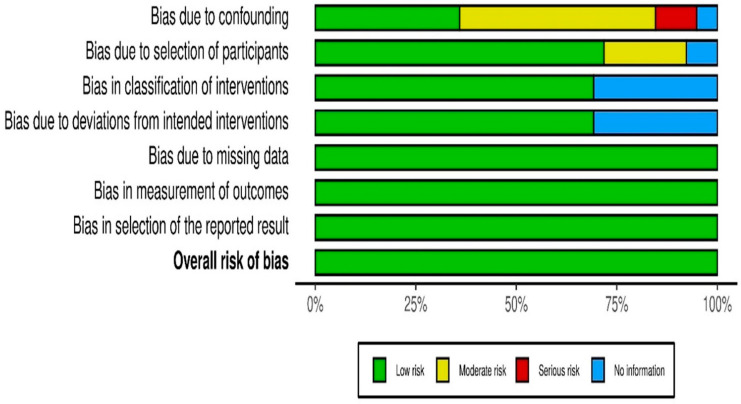
Risk of bias among the selected studies.

Cochrane Collaboration’s tools were employed [43]. The authors examined the selected studies regarding confounding, participant selection, interventions, missing data, outcome measures, and selection of the reported result to determine whether the risk of bias in studies was low, moderate, serious, or lacking information. The risk of bias in non-randomized studies of interventions (ROBINS-I) tool was used as a part of GRADE’s certainty rating process for observational studies [21,44]. The implementation of GRADE with ROBINS-I provided a common metric to assess risk of bias in RCTs and NRSs. All subcategories were equally weighted in the overall quality assessment results. The results of the grading of evidence quality and risk of bias were compared, and then generated as Figure 2 via the Risk-of-bias VISualization platform [45].

### 2.5. Data Analysis and Synthesis

#### 2.5.1. Data Analysis Procedure

The review management software Comprehensive Meta-Analysis (version 3.3; Biostat, Englewood, NJ, USA) and SPSS (version 27.0, IBM Corp., Armonk, NY, USA) were applied to manage and generate research data. The meta-analysis involved four steps: First, data entry, obtain correlations between PA and ecological factors along with sampling error variances; second, data screening, screen for outliers, which were defined as correlations whose residuals had z-scores either greater than 3.29 or less than −3.29, and exclude these [42]; third, estimate the overall effects and heterogeneity; last, perform publication-bias analyses. To ensure appropriate weighting of each individual study included in the meta-analysis, studies contributing multiple outcomes were aggregated such that each one contributed a single effect size to the analysis [30]. Studies with more than one correlation (e.g., parental PA behaviors and home environments) were treated as separated studies with individual inputs (e.g., first input in CMA is parental PA behavior and participants’ PA; the second is home environments and participants’ PA). The decentering of studies resulted in a clearer demonstration of the correlations and reduced analysis meta-biases [46,47].

#### 2.5.2. Data Synthesis

Further, the authors categorized all articles and extracted data from the final pool. Pearson’s *r*, means, standard deviations (SDs), sample size, *F*-tests, *t*-statistics, and *p* values were extracted; where these were unavailable, other relative statistics were extracted (e.g., odds ratios) to calculate effect sizes (*r*), 95% confidence intervals, and standard errors (SEs) for PA participation and influential factors. To arrive at these aggregated outcomes, each influential factor was treated individually. For instance, if a study included two influential factors and two PA outcomes, two effect sizes were calculated for that study. Then, in the overall effect size aggregation, these two effects were combined with all other outcomes. All effect sizes were converted to Fishers’ *z* and then calculated as a summary correlation ′*r* to allow comparisons across studies. The effect size was calculated via comprehensive meta-analysis through the synthesis approach of transferring individual correlation to Fisher’s *z* and conversing back to summary correlation ′*r* [46,48,49].

#### 2.5.3. Heterogeneity and Publication Bias

Finally, the heterogeneity test between studies was assessed regarding the *Q* statistic and the *I²* statistic. Statistical heterogeneity was assessed using the *p* value, *Tau*^2^, and *I*^2^; and *I*^2^ of 25% was considered low, 50% was moderate, and 75% or more was high [30]. Sensitivity analysis was applied to further reduce heterogeneity by removing studies with a high risk of bias. Lastly, the funnel plot and the Egger test were employed to assess the publication bias.

### 2.6. Subgroup Analysis

Subgroup analyses were performed on gender and variant ecological systems. The intention was to examine and specify the effect of the ecological systems or factors on non-organized PA participation in children and adolescents.

### 2.7. Data Visualization

Co-occurrence analysis was used to identify popularity themes and the research trend across selected studies via VOSviewer (version 1.6.16; [50]). This process helped to visualize the multi-dimensional interconnections among selected studies and justify the credibility of the theoretical framework in the current study. The co-occurrence analysis was calculated on a co-occurrence matrix and the construction of a map was a process included: primarily, a similarity matrix was calculated based on the co-occurrence matrix (see Supplementary Materials File B). Then, a map was constructed by applying the VOS mapping technique to the similarity matrix, which was weighted on total link strength. Lastly, the map was translated, rotated, and reflected to illustrate the themes and trends [50].

## 3. Results

Thirty-nine empirical studies were selected. Altogether, 85 correlates were identified, which were consistently associated with PA of children and/or adolescents: heritability, sex, age, PA enjoyment, gene-environment, parental and peer PA, family income, socioeconomic status (SES), perceived competence, self-efficacy, goal orientation/motivation, perceived barriers, participation in community sports, parental support, support from significant others (i.e., peers, friends), neighborhood safety, access to sport/recreational facilities and weather, and so on, which were categorized as individual (demographic, psychological, and behavioral factors), microsystem (peers, family, school, and neighborhood), exosystem, macrosystem, and chronosystem within ecological frameworks. The reference codes of the selected studies are listed in Table 2.

**Figure 3:**
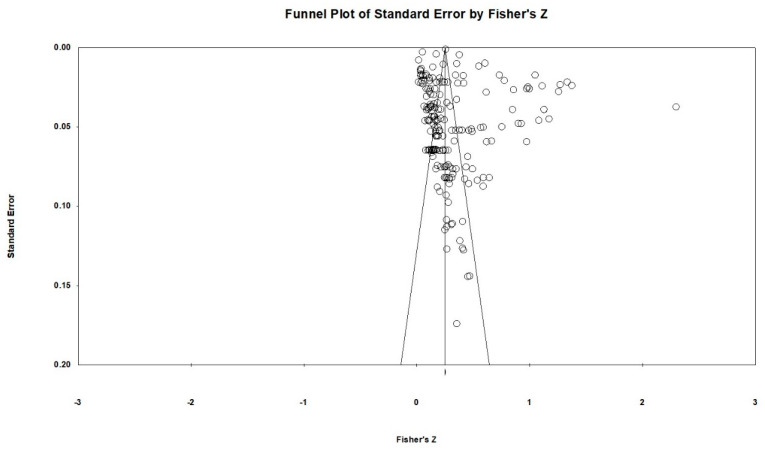
Funnel plot of publication bias.

Descriptive statistics of the countries, ecological systems, participants’ age, and research designs in the selected studies are depicted in Table 2. The median publication year of these studies was 2015 (mean = 2013.8; *SD* = 5.05; range = 2003–2021). These studies (*N* = 324,953, the total number of effect sizes *k* = 195) had a mean total sample size of 8832.13 (*SD* = 30,525.73; range = 88–138,014) and the median age of participants was 11.74 (mean = 11.75; *SD* = 4.12). Overall risk of bias was low, and a graphical display of the ratings is depicted in Figure 2. Publication bias was assessed using SEs and Fisher’s *z* in the funnel plot (see Figure 3).

### 3.1. Influence Factors

Influence factors within ecological models are discussed in the following order: individual, microsystem, exosystem, macrosystem, and chronosystem factors. Finally, co-occurrence analysis outcomes are reviewed.

#### 3.1.1. Individual

The individual is positioned at the center of the concentric circles in the ecological framework [26,61,75]. Nineteen studies discussed the correlations between individual factors and PA participation. Individual factors included the physical/demographic [8,38,54,57,59,61,62,63,67,68,69,75,76,80,81], psychological [8,57,59,62,75,79], and behavioral [8,9,54,56,57,59,62,63,68,69,73,75,76,79,80,81] characteristics of children and adolescents. 

**Demographic Factors**. Results revealed that adolescent girls tended to spend less time in non-organized PA compared with boys [38,62,75], who had higher initial levels of PA than girls [54]. One study disclosed girls were twice as likely to be inactive [63]. Adolescent PA declined significantly from ages 12 to 17 [8,54]. Across genders, greater baseline MVPA and early PA habits remained significantly associated with MVPA at follow-up, and are the most important predictor of PA levels in adolescents [57]. Conversely, having a low cardiorespiratory fitness and lower body muscular fitness increased the risk of having low intention to be physically active in adolescence [80]. In addition, family income was a significant predictor [67], but the effect was conditional on both the type of activity, organized versus discretionary, and more dependent on gender. 

**Psychological Factors.** The likelihood of participating in non-organized PA was significantly predicted by enjoyment of PA [8,75,79]. Self-efficacy was positively associated with PA in boys [62] and greater self-efficacy remained significantly associated with MVPA at follow-up [57]. In girls, efficacy to overcome barriers significantly predicted MVPA [54,57]. Lastly, consistent predictors across multiple PA measures were having appropriate apparel or accessories for PA participation [8].

**Behavior Factors.** Results supported associations between individual factors and self-reported PA as theorized within the ecological frameworks [61]. Specifically, children’s independent mobility (CIM) could be one such determinant of PA. One of the studies [73] provided several insights into this, which introduced active to school (ATS) as an effective approach to promote PA in adolescents. Individual correlates, including children’s grade, language spoken at home, car ownership, and phone ownership, were associated with CIM, which associated with PA [68]. It is worth noting that the mix of individual characteristics and social correlates and behavior showed the strongest association with adolescents’ MVPA [59]. Sex differences existed among the individual and behavioral factors related to PA and screen-based behaviors [9,62]. Having a low PA level and more screen time increased the risk of having low intention to be physically active in adolescents [80]. 

#### 3.1.2. Microsystem

Microsystem included four categories of influential factors, which were most frequently discussed in selected studies [3,20,64]. Family [5,6,9,38,51,52,53,55,58,59,63,64,66,70,71,74,75,76,77,78,81], peers [5,52,54,55,56,58,59,60,62,69,75,77,79,81,82], school [59,61,63,65,76], and community [7,51,59,61,63,67,76,79,81] were included within the microsystem. 

**Family Influence.** Family support was associated with unstructured active play across genders [51]. Combined with demographic characteristics, boys reported greater social support than girls in both family and social perspectives [52]. A warm and supportive psychosocial home environment in childhood and adolescence increased not only the mean level of subsequent PA time but also modified the genetic and environmental variances in childhood and adolescence [74]. Parenting skills and health behaviors differentiated by five overarching constructs, including child PA [6]. A physically active parent was significantly associated with girls’ and boys’ PA and/or sports participation. Parents’ gender was found to be a strong factor in children and adolescents’ PA participation [8,53,71,78]. In addition, parental modeling of PA before adolescence and logistic support during adolescence could help girls establish early patterns of PA habits [5], and girls who perceived a more supportive home PA environment were more physically active [62]. Meanwhile, perceived benefits of PA differed significantly based on whether the respondent received parental encouragement and had a friend who exercised [58]. Parental co-participation increased adolescents’ PA level [66] and reduced screen-based behaviors [62], and adolescents who received parental encouragement regarding exercising engaged in significantly more days of PA [58]. Socioeconomic status, body weight, parental education backgrounds, and parental-reported barriers were significantly associated with frequency of participation in both extracurricular exercise and PA [70]. A significant predictor of decline in spontaneous PA was family socioeconomic position [64,75]. Children of parents with more education participated significantly more in PA and/or sports than those of parents with less education [38,63]. Lastly, siblings who encouraged, co-participated, watched, talked with, and provided transportation contributed to increased adolescent PA participation [55]. 

**Peer Influence.** PA participation in children and adolescents tended to be influenced by their social environments [3,75]. Greater levels of friends’ PA, especially MVPA, were associated with increased levels of objective PA in adolescents [54,56,79]. Friendships were more likely among adolescents who engaged in greater PA and who were similar to one another in body mass index (BMI) and PA participation [58,60], and significant similarities were found between reciprocal best friend dyads [69]. Additionally, best friend’s gender, perceived exercise barriers, and social support were significant correlates of PA in children and adolescents [59,69,82]. Adolescents with higher perceived social support from friends and social networks demonstrated higher levels of PA participation [5,77], and boys garnered greater social support than girls [52]. However, girls who perceived more peer support were more physically active than boys [62]. In addition, friends who supported and engaged with or watched were significantly and positively related to PA in children and adolescents [55]. 

**School Influence.** School PA promotion programing significantly increased students’ weekday and weekend MVPA [65]. Specifically, schools that hosted one to two sport meetings annually and provided accessible sport facilities were positively correlated with student PA participation [63,69]. Children and adolescents who participated in school sport teams reported higher PA participation than their counterparts [62]. Within the childhood group, preschoolers’ participation in organized sports was positively correlated with MVPA (on weekends) and total PA [76]. Lastly, after-school activity functioned as a means of gender socialization, which remained as a predictor of children’s PA [64]. Although teacher and coach influences have been considered a vital part of schools’ influence, there was limited evidence in the current literature [83].

**Community Influence.** Difficulty regarding the accessibility of community recreational facilities and concerns of neighborhood safety were negatively associated with children and adolescents’ PA participation. Instead, higher safety reported neighborhood was positively associated with spontaneous active play across genders [51,63]. The accessibility to public recreation facilities and the location were significantly associated with PA in girls [81]. Environmental opportunities were associated with change in PA in adolescents [79]. Neighborhood household income level was a significant predictor of both sports and PA and active free play [67]. Adolescents who lived in neighborhoods without sidewalks were 1.3 times more likely to be inactive than counterparts [63]. 

#### 3.1.3. Exo-, Macro-, and Chronosystems

Exo-, macro-, and chronosystem included influence factors of extended family, public policy, culture, and economy [19,23,26,84,85]. Three systems’ factors of policy, culture, and economy [7,38,39,59,64] and weather [7,72] were reviewed altogether because those systems were deep-rooted with each other in selected studies. Differences in the physical, socio-cultural, sport policy, economic environment, and gross domestic product (GDP) among countries were reported as significant predictors in adolescent PA [38,39,59]. Through a lens of culture [64,70], PA was greatly gendered under the Asia cultural context. Moreover, greater urbanization, higher adult PA, and higher average national household income were positively correlated with adolescent VPA [7]. Ambient temperature and rainfalls [72] had substantial effects on children’s daily step counts and should therefore be considered when comparing PA across different regions or seasons. Noteworthy, lower temperatures were associated with more MVPA in Europe [7]. 

#### 3.1.4. Differences between Children and Adolescents

PA participation patterns and influential factors in studies were different due to participants’ age, which were basically categorized as children and adolescent cohorts. In studies that included children, parental support [5,6,53,70,71,76,78], and neighborhood environments [8,62,68] were dominant influential factors in children’s non-organized PA participation. Among studies with adolescent participants, PA participation was affected by more internal factors: PA enjoyment and self-efficacy [56,59,74,80] and social interactions [52,54,55,58,60,64,69,77]. As age increased, PA participation exhibited complexities in both external and internal patterns in adolescents’ PA participation, especially during the transition of puberty. Meanwhile, gender [67], family support [51,57,63,67,73,75], and macro environments [38,39,72,81] continuously affect PA participation across childhood and adolescence.

### 3.2. Aggregate Correlations

The aggregate random-effects effect size and meta-analytic average of the correlations was (*′r* = 0.32, 95% CI 0.30 to 0.34, *k* = 195, *p* < 001). This revealed that ecological factors could have a moderate positive effect on children and adolescents’ PA participation. The *Q*-statistic evaluated that the variability in the aggregated effect sizes was significant (*Q* = 33,810.25, *p* < 0.001, *I*^2^ = 99.43%, *Tau*^2^ = 0.022), indicating that there was significant variability among the effect sizes. This suggested that the planned follow-up moderator analysis is warranted, as analyzed in different subgroups. A classic fail-safe N calculation indicated that 3663 null studies would be required to change the current findings from significant to nonsignificant. This yielded confidence that the significant aggregated effect was unlikely to be spurious or inflated as a result of an abundance of unpublished null results. Aggregate results from subgroup analysis indicated that theory-based influence factors achieved moderate effect with boys (*′r* = 0.37, 95% CI 0.24 to 0.48, *k* = 40, *p* < 0.001) and girls (*′r* = 0.32, 95% CI 0.22 to 0.42, *k* = 55, *p* < 0.001) in PA participation. Interestingly, moderate strong correlations were found between ecological factors and twins’ PA participation (*′r* = 0.61, 95% CI 0.30 to 0.80, *k* = 3, *p* < 0.001) and heterogeneity (*Q* = 3.23, *p* = 0.20, *I*^2^ = 98.94%, *Tau*^2^ = 0.17). In general, individual (*′r* = 0.32, 95% CI 0.23 to 0.41, *k* = 66, *p* < 0.001), macro-, and chronosystems factors (*′r* = 0.50, 95% CI 0.46 to 0.53, *k* = 18, *p* < 0.001), such as age, gender, psychological and behavioral characteristics, weather, national-level economy, safety environment, and sport policy, appeared more influential than microsystems factors (*′r* = 0.28, 95% CI 0.24 to 0.33, *k* = 111, *p* < 0.001) on children and adolescents’ PA participation and engagement. 

**Figure 4:**
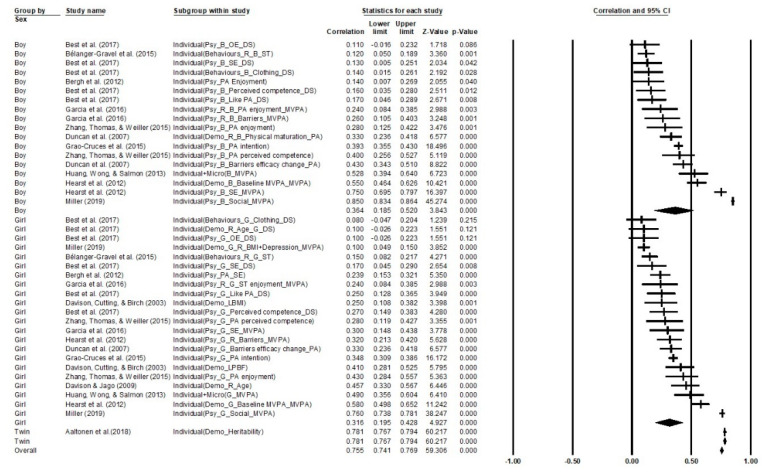
Correlations on individual factors in children and adolescents’ PA. Note. ASP = After school physical activity promotion; ATS = Active transport to school; B = Boy; CIM = Children independent mobility; DAP = During adolescent period; DS = Daily steps; R = Reversed correlation; EO = Environmental opportunities; ES = Extracurricular sport; G = Girl; OE = Outcome experiences; PD = Parent daughter; SE = Self-efficiency; SPE = School physical education promotion; WD = Weekend; WY = Weekday.

**Figure 5:**
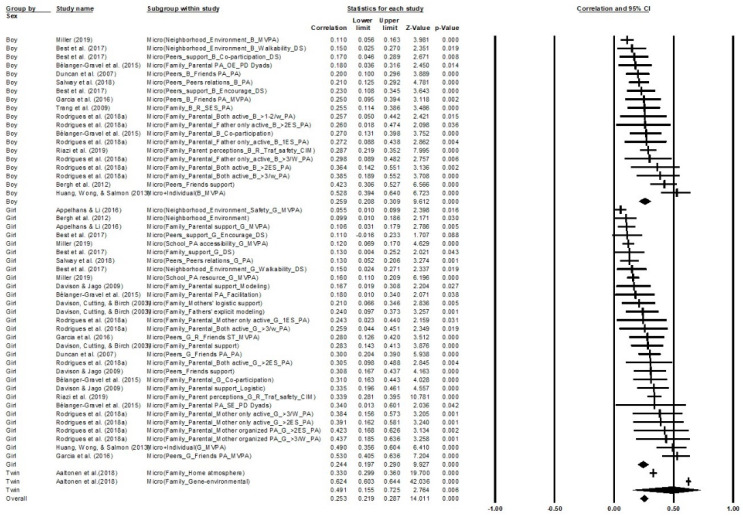
Aggregate correlations on microsystem factors in children and adolescents’ PA. Note. ASP = After school physical activity promotion; ATS = Active transport to school; B = Boy; CIM = Children independent mobility; DAP = During adolescent period; DS = Daily steps; R = Reversed correlation; EO = Environmental opportunities; ES = Extracurricular sport; G = Girl; OE = Outcome experiences; PD = Parent daughter; SE = Self-efficiency; SPE = School physical education promotion; WD = Weekend; WY = Weekday.

**Figure 6:**
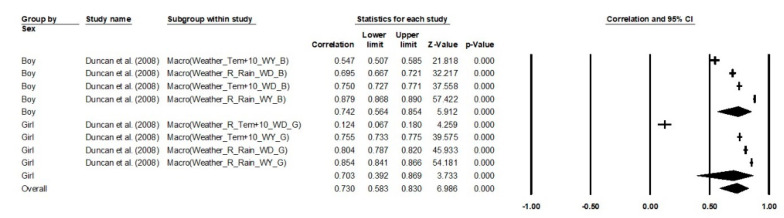
Aggregate correlations on macrosystem factors in children and adolescents’ PA. Note. ASP = After school physical activity promotion; ATS = Active transport to school; B = Boy; CIM = Children independent mobility; DAP = During adolescent period; DS = Daily steps; R = Reversed correlation; EO = Environmental opportunities; ES = Extracurricular sport; G = Girl; OE = Outcome experiences; PD = Parent daughter; SE = Self-efficiency; SPE = School physical education promotion; WD = Weekend; WY = Weekday.

Further, subgroups of studies were analyzed in two clusters: the first cluster of three models were categorized by each ecological system, and then boy, girl, and twin samples were subgroups within the models. The other three mix gender models were categorized by ecological systems (see Figure 4, Figure 5, Figure 6). Gender differences on individual factors were significantly correlated with children and adolescents’ PA participation; boys (*′r* = 0.36, 95% CI 0.19 to 0.52, *k* = 17, *p* < 0.001) showed a higher moderate correlation with individual factors than girls (*′r* = 0.32, 95% CI 0.20 to 0.43, *k* = 21, *p* < 0.001). Intriguingly, twins’ PA participation was significantly correlated with individual factors (*′r* = 0.78, 95% CI 0.77 to 0.79, *k* = 1, *p* < 0.001) and was higher than both single gender subgroups. The overall correlation was strong, which showed that individual factors could significantly promote children and adolescents’ PA participation across all gender groups (*′r* = 0.76, 95% CI 0.74 to 0.77, *k* = 39, *p* < 0.001) and the quality of heterogeneity across samples was satisfied (*Q*_Total between_ = 147.56, *p* < 0.001, *I*^2^ = 98.72%, *Tau*^2^ = 0.16).

Gender differences on microsystem factors were significantly correlated with children and adolescents’ PA participation; boys (*′r* = 0.26, 95% CI 0.21 to 0.31, *k* = 18, *p* < 0.001) disclosed a higher small correlation with individual factors than girls (*′r* = 0.24, 95% CI 0.20 to 0.29, *k* = 30, *p* < 0.001). Similar to individual factors, twins’ PA participation was also significantly moderate strong correlated with micro factors (*′r* = 0.49, 95% CI 0.16 to 0.73, *k* = 2, *p* = 0.006) and was higher than both single gender subgroups. The overall correlation was small, which showed that micro factors could significantly promote children and adolescents’ PA participation across all gender groups (*′r* = 0.25, 95% CI 0.22 to 0.29, *k* = 50, *p* < 0.001) and the quality of heterogeneity across samples was satisfied (*Q*_Total between_ = 2.249, *p* = 0.33, *I*^2^ = 95.10%, *Tau*^2^ = 0.05).

Gender differences on macro- and chronosystems factors were significantly correlated with children and adolescents’ PA participation; boys (*′r* = 0.74, 95% CI 0.56 to 0.85, *k* = 4, *p* < 0.001) showed slightly higher strong correlation with macro- and chronosystems factors than girls (*′r* = 0.70, 95% CI 0.39 to 0.87, *k* = 3, *p* < 0.001). The overall correlation was moderate strong, which showed that macro- and chronosystems factors could significantly promote children and adolescents’ PA participation across all gender subgroups (*′r* = 0.73, 95% CI 0.58 to 0.83, *k* = 7, *p* < 0.001) and the quality of heterogeneity across samples was satisfied (*Q*_Total between_ = 0.08, *p* = 0.77, *I*^2^ = 99.53%, *Tau*^2^ = 0.14). Noteworthily, the correlations of macro- and chronosystems were higher than individual and microsystem factors, which might be due to the limited number of studies within this category. There were no data related to the twins’ sample and exosystem included in this cluster.

In the mixed gender models, individual factors showed significantly small to moderate correlation with children and adolescents’ PA participation (*′r* = 0.30, 95% CI 0.17 to 0.42, *k* = 24, *p* < 0.001) and the quality of heterogeneity across samples was satisfied (*Q*_Total between_ = 7808.31, *p* < 0.001, *I*^2^ = 99.64%, *Tau*^2^ = 0.14). Microsystem factors showed significantly small correlation with children and adolescents’ PA participation (*′r* = 0.28, 95% CI 0.23 to 0.33, *k* = 63, *p* < 0.001) and the quality of heterogeneity across samples was satisfied (*Q*_Total between_ = 2845.14, *p* < 0.001, *I*^2^ = 98%, *Tau*^2^ = 0.05). Lastly, macro- and chronosystems factors showed significantly small to moderate correlation with children and adolescents’ PA participation (*′r* = 0.33, 95% CI 0.29 to 0.37, *k* = 11, *p* < 0.001) and the quality of heterogeneity across samples was satisfied (*Q*_Total between_ = 8982.78, *p* < 0.001, *I*^2^ = 99.88%, *Tau*^2^ = 0.007).

### 3.3. Outcomes of Co-Occurrence Analysis

**Figure 7:**
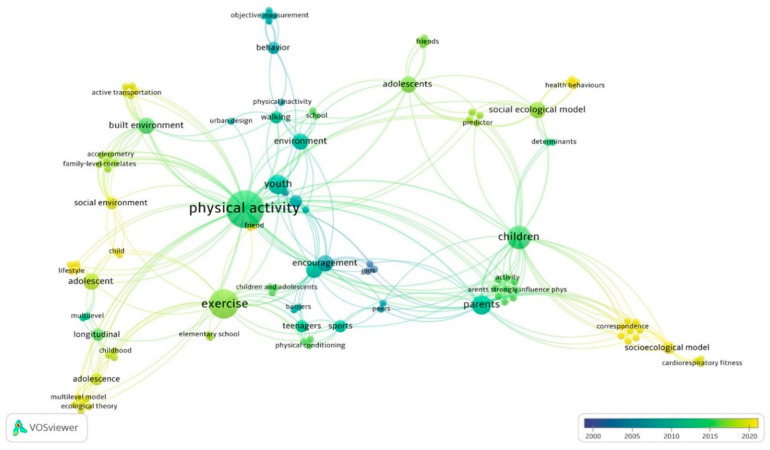
Visualization of co-occurrences and keywords in the selected studies.

The co-occurrence analysis was conducted based on selected studies (see Figure 7). It depicted the core of the themes and keywords in children and adolescent research with a concentration in PA, exercise, and health. The color scale transitions from dark to light, demonstrating the trend of the selected studies in the last two decades. Shifting our focus to the edge (see Figure 7) showed that ecological theories and frameworks have become a hot spot in the past few years. Further, the results in the co-occurrence analysis demonstrated from the other side that the ecological framework was eligible and aligned with the purpose of the study.

## 4. Discussion

The intention of this study was to examine promoting/inhibiting correlates associated with PA and further demonstrate the complexity of spontaneous PA participation in children and adolescents using an ecological framework. Therefore, we explored the following research questions: First, what are the promoting/inhabiting factors that significantly impact children’s PA participation, and which is the most influential factor for children and adolescents’ PA within the ecological system? Second, what are the limitations regarding the design, sampling, and measurements in the current selected studies? Last, what is the current trend and future direction of this research area? The systematic review and meta-analysis revealed that the overall ecological systems significantly and moderately correlated with children’s and adolescent’s PA. Results from subgroup analysis showed that theory-based influence factors achieved a moderate effect with boys and girls in PA participation. The meta-analytic average of the correlations between ecological factors and twins’ PA participation was found to be higher than others. Noteworthily, exo-, macro-, and chronosystems factors (e.g., weather, geographic, and policies) were more influential than microsystem factors (e.g., parental encouragement, support, and co-participation; peer social networks, support, and enjoyment) and individual factors (e.g., behavioral, demographic, and psychological) in promoting children and adolescents’ PA participation. Ecological system factors and limitations in the selected studies are discussed in the following sections.

### 4.1. Individual Characteristics and Peer Influences

Similar to parental support, friend MVPA appeared to have the greatest association with both boys’ and girls’ objective MVPA [56]. Peer relations theory and research indicate that same-age peers provide unique opportunities for companionship and recreation during childhood and adolescence [86]. Peers’ co-participation in activities and modeling of PA are the primary mechanisms that drive this association [56]. Many physical activities during these developmental periods typically involve some form of play, whether organized sports or spontaneous daily active play, that requires play partners [87,88]. Therefore, it should not be too surprising that a number of empirical studies [5,52,54,55,56,58,59,60,62,69,75,77,79,81,82] have found that children and adolescents are more physically active when in the presence of peers than when alone. Children and adolescents who reported a greater presence of peers in their lives also reported greater PA participation, which corresponded to previous evidence that lonely children, who were often friendless and rejected by peers, reported the least amount of PA [53,89,90,91]. Increasing positive feedback and support from peers might prove advantageous in improving activity levels in children and adolescents [52]. Peers could also increase the variety of physically active alternatives, which in turn had been shown to increase the amount of PA participation and their enjoyment of that activity or sport [92]. Moreover, increasing individual physical fitness and life satisfaction [9,80,82] as well as reducing screen time could also positively influence the intention of adolescents to be physically active. Overall, the existing works strongly suggested a peer is a necessary element for PA during childhood and adolescence.

In terms of peer influences on PA, positive associations have been shown between friend support and PA in adolescent [93]. In adolescent girls, PA might be facilitated by the development of new social networks, and peer support was found to be very important in maintaining PA [94]. Barriers to PA in adolescent girls included negative experiences because of a school PE curriculum that emphasized muscularity [94]. Negative PE class experiences could relate to social interactions, and concerns around appearing too muscular could be linked to norms regarding the socially accepted feminine body shape.

### 4.2. Family and Macrosystem Influences

Parental support has been shown to be a positive predictor of PA in children and adolescents [95]. Positive correlates were shown among parental encouragement, support, modeling, and attitudes towards PA for adolescent PA participation; however, it was questionable due to opposing findings [93,96]. A study [53] argued that parents could have positive contributions to young daughters’ activity practices. Promoting co-participation in PA and less sedentary activities appear as useful targets for increasing PA among parents and tweens [66]. PA parenting, defined as parental behaviors intended to influence children’s PA, may positively influence children’s PA, although the conceptualization and measurement of the construct of PA parenting have been criticized [97]. Parental support, along with higher household income and higher levels of parental education, has been associated with increased PA in adolescents [94,98]. Siblings’ PA was found to increase adolescents’ PA participation [95]. Additionally, the family environment is critical for not only youth development, but also PA participation and engagement [74]. The interplay between the genes and psychosocial home environment in childhood and adolescence seems to be important when explaining differences in leisure time PA behavior in young adults. Positive social interactions at a neighborhood level might be conducive to adolescent PA participation [99]. PA interventions for low-income families are needed in practice and efforts in support of urban children should emphasize unstructured active play, particularly in boys [51]. 

At the macrosystem level, Norway acted as an extraordinary example of the effectiveness and application of ecological systems to positively influence childhood and adolescence PA and exercise [100]. Despite the weather conditions [7,72], an abundance of natural and artificial athletic facilities alongside a well-established voluntary sports club distribution and a scientific-based school PE system, and high levels of parental involvement reinforced PA in children and adolescents. Moreover, the favorable country-level economic condition also created the optimal environment for children and adolescents’ PA participation [101]. All these factors were deeply embedded in Norwegian society and strengthened the physical recreation cultures. In terms of some experiences used for references for policy towards PA and PE beyond Norway, there might be grounds for some optimism around parental involvement in active play as well as the effectiveness of peer support of PA participation. However, the correlations between exo-, macro-, and chronosystems factors with PA appeared higher than other systems, possibly because of the heterogenous evidence within selected studies. Therefore, future research (more feasible and longitudinal designs, such as applying the PPCT model) is encouraged to shed light on big environment factors (e.g., weather, geographic, policies, and sociohistorical) to explicate influences on PA in children and adolescents.

Exploring the interactions among multiple systems and PA was imperative when grounded in ecological frameworks. According to SEM, children’s behavior stemmed from reciprocal interactions among micro-, meso-, exo-, and macro-systems [102]. While focusing on gender differences, effective PA promotion strategies should integrate individual-, family-, school-, and community-level factors altogether [63]. Thus, to be able to make substantial behavioral changes, future PA promotion strategies must recognize children and adolescents’ PA participation as a dynamic system and target changes at each level of the ecological framework.

### 4.3. Limitations and Future Directions

#### 4.3.1. Research Designs

Ecological theory and systems have an interactive and cumulative effect on change [3]. Therefore, a longitudinal or experimental research design is encouraged in future studies to address a critical need for data on patterns of adolescent PA and how it changes over time and further determine if improvements in the constructs of interest were related to PA behavior changes [103]. As correlational research cannot conclude causality, it would be necessary to conduct more random control trials (RCTs) in the future to provide more rigorous evidence to the field. Future RCTs should be mindful of delivering evidence-based interventions that target the long-term effects of ecological factors on participating in PA. Study designs are depicted in Table 2.

#### 4.3.2. Measurements

One of the major limitations of the selected studies was the self-reported PA, which might imply a potential bias of subjectivity. In 26 studies, the measures were based on adolescents’ self-report regarding yes or no questions and secondary data [6,7,38,39,52,53,55,58,59,60,61,62,63,64,66,67,68,69,70,71,73,74,75,77,78,80,81], which might be one potential limitation of this study. Self-reports have several shortcomings with respect to validity and reliability in terms of recall and social desirability biases. Although self-reported height and weight are highly correlated with the actual number, it would still be important to employ objective PA assessment, such as accelerometers, pedometers, and heart rate monitors, to increase the validity and reliability of the data in future studies. Additionally, studies underestimated parental contribution in that some domains of parental support were not assessed. For instance, the extent to which parents restructured their home environment to make it less conducive to sedentary activity [9,38,65,66,80,82]. Besides, a low completion rate [38,39] and lack of assessing pubertal status [57] should also be reconsidered in future studies.

#### 4.3.3. Sampling

The majority (96.8%) of participants were white and recruited from urban areas in Western countries. The imbalance of gender and ethnicity limits the generalizability of the results [57], restricting the ability to make comparisons by race [5,6,8,55]. Moreover, only positive supportive behaviors were examined, which limited the ability to make inferences on the influences of negative behaviors (e.g., teasing, laughing at activity attempts) on activity levels [8]. Furthermore, social network techniques were susceptible to bias because missing data impacted the structure of the network [60,69]. Although limitations were detected in the selected studies, the confidence in the conclusions from the current study was moderate to strong based on the adequate sample size (*N* = 324,953) and heterogeneity of studies among 41 countries. The synthesis data bolstered the weakness within individual studies and increased sample diversity. Future studies are encouraged to focus on marginalized populations, for example, people of color, adolescent females, and immigrant families from different cultural backgrounds. 

#### 4.3.4. Future Directions

To the best of our knowledge, the number of children participating in organized sport activities (e.g., sport clubs, teams) has increased in the last decades while non-organized or spontaneous PA, even overall PA participation, has decreased significantly over time [104,105]. Since the unrepresentative population appeared more engaged in non-organized PA participation, this trend undoubtedly exacerbated the polarization [75]. Hence, in the post pandemic period, the questions of how to motive more children and adolescents back to the playground and sport facilities should be considered as the priority public health topic. Children and adolescents should accumulate a minimum of 60 minutes of MVPA daily as part of transportation, PE sessions, sport, free play, and planned exercise [11]. Considering the small percentage of children and adolescents who met the WHO and CDC guidelines prior to the global pandemic, encouraging and guiding them to engage in physical activities is even more important in the current circumstance. Additionally, future research is encouraged to fill the gap on non-organized sport to further explore the relationship among ecological factors and PA participation, for example, recreational tennis, cycling, or swimming, in children and adolescents. Metcalf and colleagues [33] provided strong evidence that PA interventions have had only a small effect (approximately four minutes more walking or running per day) on children’s overall activity levels. This finding helped to explain, in part, the reason such interventions had limited success in health promotion, such as reducing BMI or body fat in children. Further, compared to organized PA, the long-term effects of lasting non-organized PA participation in children and adolescents (e.g., habit formation, PA self-efficacy, etc.) are still uncertain. 

## 5. Conclusions

Predicting PA during childhood and adolescence is a complex topic [26,61,106]. The current study provided evidence that children and adolescents’ non-organized PA participation is significantly correlated with multiple layers of ecological factors. The likelihood of participating in non-organized PA in children and adolescents was significantly predicted by sex, age, enjoyment of PA, self-efficacy, outcome expectations, socioeconomic status, parental support (e.g., co-participation and encouragement), peer influences (e.g., play-with and support, etc.), and proximal environments (e.g., home, neighborhood, and safety levels, etc.). Girls tended to spend less time in non-organized PA across all age groups, compared with boys, and as age increased, PA participation dropped. Noteworthy, exo-, macro-, and chronosystems included the most influential factors correlated with PA participation in children and adolescents. Potential strategies to promote non-organized PA participation in children and adolescent might include an inclusive environment for girls and minoritized groups, emphasizing the benefits of non-organized PA effectively decreasing screen time and sedentary behaviors, and building an active family environment. Furthermore, PA is a spectrum that is grounded in a diversified cultural phenomenon and different sports or physical activities are enjoyed by specific populations and age groups, even countries. Therefore, future research is encouraged to add specificity to the long-term effects of lasting non-organized PA participation in children and adolescents. Lastly, interfering with the global pandemic, the question of how to effectively motivate children and adolescents back to physical and social activity is considered as the priority task in the post-COVID-19 era.

## Figures and Tables

**Table 1:** Search strategy.

Concept	Keywords
I. Outcome: PA participation and engagement	^1,3,5^ participation or ^2^ physically active or ^2^ or ^1,4^ leisure activity [MeSH Terms] or ^1,4^ execrise [MeSH Terms] or ^1–7^ physical activity or ^1–4,7,8^ vigorous physical activity or ^1–4,7,8^ moderate to vigorous physical activity or ^1–4,7,8^ light physical activity or ^1–4^ sedentary behaviors or ^1,4^ BMI [MeSH Terms] or ^1–4,7,8^ self-efficacy.
II. Participants: Youth population (Age < 18)	^1–8^ youth or ^1–8^ adolescent * or ^1–7^ young people or ^1–7^ teen or ^1–7^ young adult * or ^1–7^ children or ^1–7^ kid * or ^1–8^ teenager *.Exclude (NOT): ^1–4,6–8^ smoking or ^1–4,6–8^ drinking or criminal * or ^1–4,6–8^ disability or ^1–4,6–8^ injury or ^1–4,6–8^ handicap or ^1–4,6–8^ disorder or ^1–4,6–8^ resistance training or ^1–4,6–8^ injuries or ^1–4,6–8^ accident or ^1–4,6–8^ trauma or ^1–4,6–8^ older adult * or ^1–4,6–8^ elderly or ^1–4,6– 8^ seniors or ^1–4,6–8^ geriatric *.
III. Exposure: Influence factors	^1–8^ Family or ^1–8^ peer * or ^1–8^ community or ^1–8^ school or ^1–8^ coach or ^1–8^ teacher or ^1–8^ social context or ^1–8^ social-economic or ^1–8^ socioeconomic status or ^1–8^ friend * or ^1– 8^ individual * or ^1–8^ policy or ^1– 8^ cultur * or ^1–4,6–8^ health promotion interventions or ^1–8^ physical education session *.
IV. Exposure: Exercise types	^1–8^ physical exercise or ^1–8^ exercise * or ^1–8^ fitness or ^1–8^ physical activity or ^1–8^ sport* or ^5^ dance or ^1–8^ walking or ^1–8^ non-organization sport * or ^1–8^ recreational sport * or ^1–8^ motor activity or ^1–8^ leisure activity.
V. Theoretical Framework	^1–4,6–8^ Ecological theory or ^1–4,6–8^ ecological framework or ^1–4^ PPCT or ^1–4,6–8^ social ecological or ^1–4,6–8^ social ecological theory or ^1–4,6–8^ socioecological theory or ^1–8^ socioecological framework or ^1–4,6–8^ socioecological *.
V. Design	^1–4,6–8^ experimental study or ^1–4,6–8^ experimental research or ^1–4,6–8^ quasi experimental study or ^1–4,6–8^ empirical study or ^1–4,6–8^ quantitative study or ^1–4,6–8^ longitudinal study or ^1–4,6–8^ cross section * or ^1–4,6–8^ mixed-method or ^1–4,6–8^ social network analysis or ^1–4,6–8^ random control trail or ^1–4,6–8^ cluster random control trail or ^1,4^ RCT or ^1,4^ clinical trial or ^1,4^ treatment outcome.

Note: Databases: ^1^ PubMed, ^2^ SPORTDiscus, ^3^ APA PsycInfo, ^4^ MEDLINE Complete, ^5^ Google Scholar *, ^6^ ERIC, ^7^ Academic Search Complete, and ^8^ Dimensions; Range of publishing is from 2001 to 2021. * Google Scholar was only used to cross-validate search results from bibliographic databases. The latest search through the aforementioned databases was 8 May 2021; the strategy was rigorously followed by the protocol [21].

**Table 2:** Frequencies of countries, ecological systems, age groups, and study designs across the selected studies reviewed (*N* = 39).

	Reference Numbers	No. of Studies
United States	[5,6,51,52,53,54,55,56,57,58,59,60,61]	13
China	[62,63,64,65]	4
Canada	[66,67,68]	3
Portugal	[69,70,71]	3
Europe ^a^	[7,38,39]	3
New Zealand	[72,73]	2
Finland	[9,74]	2
Australia	[8,75]	2
Germany	[76]	1
Brazil	[77]	1
Estonia	[78]	1
Norway	[79]	1
Spain	[80]	1
Vietnam	[81]	1
United Kingdom	[82]	1
Microsystem	[5,6,8,9,38,51,52,53,54,55,56,57,58,59,60,61,63,64,65,66,67,68,69,70,71,73,74,75,76,77,78,79,81,82]	34
Individual	[8,54,56,57,58,59,60,61,62,63,67,68,69,73,74,75,76,79,80,81]	20
Macrosystem	[7,38,39,59,61,63,72,73,76]	9
Exosystem	[59,61,73,81]	4
Chronosystem	[67]	1
Children ^b^	[5,6,8,9,38,51,53,61,62,66,68,70,71,72,76,78,79,82]	18
Adolescents	[7,39,52,54,55,56,58,59,60], [64] ^c^, [65,69,73,74,77,80]	16
Mixed children and adolescents	[57] (10–16 years.), [63] (11–17 years.), [67] (9–13 years.), [75] (11–13 years.), [81] (11–16 years.)	5
Cross-sectional ^d^	[6,7,8,9,38,39,51,52,55,58,59,61,62,63,66,68,69,70,71,72,73,76,77,78,80,81,82]	27
Longitudinal ^e^	[5,53,57,60,64,67,74,75]	8
RCTs ^f^	[65,79]	2
Cohort sequential	[54]	1
Mixed method	[56]	1

Note: ^a^ Europe included 7, 29, and 34 countries in three studies [7,38,39], respectively (see Supplementary Materials File C). ^b^ According to [3,40], children is defined as 3–12 years old. Adolescent is defined as 12–18 years old. Mixed indicated participants’ age ranging from 3–18 years old. ^c^ Study [78] included 11-year longitudinal data through adolescence to young adulthood. ^d^ [71] applied cross-sectional social network analysis. ^e^ [79] applied longitudinal social network analysis. ^f^ RCTs = Random control trials; [82] applied clustered RCTs.

## Data Availability

Not applicable.

## Supplementary Materials

The following are available online at www.mdpi.com/1660-4601/18/17/9080/s1, Supplementary File A−C.
