# Supplementary material for: Correlations between Physical Activity Participation and the Environment in Children and Adolescents: A Systematic Review and Meta-Analysis Using Ecological Frameworks
Source: Int J Environ Res Public Health. 2021 Aug 28;18(17):9080. doi: 10.3390/ijerph18179080 (PMC8430662; doi:10.3390/ijerph18179080)
Supplement: Supplementary file 1 [file ijerph-18-09080-s001.zip › Supplemental File A.pdf]

Supplemental Data A - Systematic Review and Meta-analysis Data

No.	Sex (%)	PA Correlation	PA Measurement	Protocol	Findings	Unique or Novel Aspects
**1.**	F=57- 58, M=43; *n*=3305	Family (Genes and Psychosocial Home Environment)	Leisure time metabolic equivalent units (MET index).	Participants were Finnish twins. The psychosocial gene and home environment was assessed.	A psychosocial home environment that is warm and supportive in childhood and adolescence not only increases the mean level of subsequent leisure time PA in young adulthood but also modifies the genetic and environmental variances in leisure time PA.	The interplay between the genes and psychosocial home environment in childhood and adolescence seems to be important when explaining differences in leisure time PA behavior in young adults.
**2.**	F=53.4, M=46.6; *n*=88	Family income, Neighborhood environment	Accelerometry (ActiGraph GT3×+)[a]	Research staff visited 88 low-income Chicago households, documented the home availability of PA equipment.	Higher reported neighborhood safety and family support for PA were associated with engagement in unstructured active play for both genders.	PA interventions for low-income, urban children should emphasize unstructured active play, particularly in boys.
**3.**	F=52.1, M=47.9; *n*=363	Family (parental) and friends support	Questionnaire (Self-reported PA)	Students completed a questionnaire assessing self-re- ported activity and social support (SS) from 3 providers: mom, dad, and peers.	Peers, transportation, and praise affected activity levels. Boys reported greater social support than girls did.	Increasing positive feedback, transportation to places to be active, and peer support may prove advantageous in improving activity levels in this age-group.
**4.**	F=48.4, M=51.6, *n*=980	Family and SES	Questionnaire (Self-reported PA)	Participants completed a questionnaire and multilevel modelling was used.	Promoting co-participation in PA and less sedentary activities appear as useful targets for increasing PA among parents and tweens.	This study examined correlates of PA using an innovative dyadic perspective and provided new insights regarding the influence of the family context on PA.
**5.**	F=47.8, M=52.2[C]; *n*=590	Individual, family, peers, and school	Accelerometry (ActiGraph GT3×+)[a]	It was designed to increase environmental opportunities for PA at school, improve social support, self-efficacy, and enjoyment in order to enhance overall level of PA	change in enjoyment, self-efficacy, perceived social support from friends and environmental opportunities were associated with change in PA in adolescents.	A significant effect was seen among girls and normal weight adolescents.
**6.**	N/A; *n*=324	Individual, interpersonal, environmental factors	Accelerometer (New Lifestyles 1000 pedometer)[d]	Children and their parents were surveyed on personal, interpersonal and environmental correlates of PA.	Consistent predictors across multiple PA measures were parent support for PA, having appropriate clothing for sport, enjoyment of PA and perceived availability of sporting clubs.	Compared correlates of PA across three measures of PA in this age group.

**7.**	F=52, M=48; *n*=700	GDP[e]	Questionnaire (PA behaviors scale)	A school-based survey, children was conducted in seven European countries using a standardized protocol.	Large differences between countries were found in PA, TV and computer time.	Girls reported lower engagement in sport across all countries. Children of higher educated parents participated significantly more in sports than those from lower educated parents.
**8.**	F=49.2-49.4, M=50.8-50.6; *n*=1805	Age, gender, household income, and time	Questionnaire (self-report PA)	Examined two outcome measures participation in organized sport and PA and active play associate with age, time, and gender.	Household income at the neighborhood level is a significant predictor of both organized sport and physical activities and active free play.	Income to be an important predictor, but the effect is conditional on both the type of activity (organized versus discretionary), and gender.
**9.**	F = 100; *n*=174	Peers' support and parental modeling and logistic support	Accelerometer (ActiGraph 7164)[f]; Activity Support Scale (ACTS)	Parental modeling and logistic support for PA were assessed at all ages; Patterns of change in parent/peer support and differences in exposure to support for girls were assessed using individual growth curve modeling.	Parental modeling of PA before adolescence and logistic support during adolescence could help girls establish early patterns of PA and social networks that facilitate maintained PA during adolescence.	Adopted a communal approach to logistical support such as car-pooling to and from activity locations or incorporating opportunities for parents to socialize, and network, while their children participate in activities
**10.**	F=100; *n*=180	Family (Parental support)	Questionnaire (self-report PA)	Measures of girls' PA included the Children's PA scale, participation in organized sports, and physical fitness.	Girls reported significantly higher levels of PA when at least one parent reported high levels of overall support in comparison to no parents.	The positive contribution that parents can have on activity practices of their young daughters.
**11.**	F=50.1, M=49.9; *n*=373	Individual (Demo and behavior), Family and peer support	Accelerometer (The Yamax Digiwalker SW-701)[g,d]	Participants were assessed annually for 4 years at the participants' homes. The youth and a parent completed surveys in the presence of trained research assistants.	Youth PA declined significantly from ages 12 to 17. Boys had higher initial levels of PA than girls. Efficacy to overcome barriers, physically active friends, and friend social support all played roles in reducing the decline in PA.	The longitudinal nature of the study addresses a critical need for data on patterns of youth PA and how it changes over time.
**12.**	F= 51.9, M= 48.1; *n*=1115	Weather (temperature, wind speed, precipitation, and duration of bright sunshine	Accelerometry (Model NL- 2000)[h]	Values of daily (7 a.m. to 7 p.m.) mean ambient temperature, mean wind speed, precipitation, and duration of bright sunshine were obtained from local meteorological stations.	Ambient temperature and rainfall have substantial effects on children's daily step counts and should therefore be considered when comparing PA across different locations or periods.	Describing the associations between weather conditions and daily PA levels in children.

**13.**	F=50.3, M=49.7; *n*=372	Family (parent and siblings) and friends support	Questionnaire (self-report PA)	A part of the longitudinal cohort-sequential study design, aim to examine the influence of social support for PA among youth.	Friends who support and watch youth engage in activities are significantly and positively related to youth PA.	Siblings (encourage, do with, watch, talk, and transport) contribute to increase youth PA
**14.**	F=49.1, M=50.9; *n*=735	Individual, Social, and cultural potential correlations, physical environmental potential	Accelerometry (Actiheart devices)[i]	Family- and community-level potential correlates of PA from different domains were subjectively and objectively assessed.	Preschoolers' participation in organized sports was positively correlated with MVPA (on weekends) and TPA (both on weekends and weekday afternoons).	Subjective traffic safety perceptions and participation in organized sports, an indicator and a result of parental support towards PA which is more proximal to preschoolers.
**15.**	F=50, M=50; *n*=240	Family (Parent support)	Questionnaire (self-report PA, Family Health Behaviors Scale (FHBS)).	A latent profile analysis was used to identify profiles among caregiver report of parenting skills; child PA, parent health behaviors, etc.	Parenting skills and child and caregiver health behaviors differentiated by five overarching constructs including child PA.	PA is influenced among multiple ecological levels through latent profile analysis.
**16.**	F=53, M=47; *n*=152	Individual (Demo and behavior) and peer support (PA and screen time)	Accelerometry (ActiGraph GT3X)[a,d]	Three steps. Random selection of students; Focus groups; Collection of accelerometers<br>Self-reported PA and screen time<br>Collection of friends.	Greater levels of friends' MVPA were associated with increased levels of objective MVPA in adolescents	Friends' MVPA appeared to have the greatest association with both male and female adolescents' objective MVPA, friends' co-participation in activities and modeling of PA are the primary mechanisms that drive this association.
**17.**	F=49.4, M=50.6; *n*=1986	Individual (Intention to be physical active)	Questionnaire (self-report PA, Health Behavior in School-aged Children (HBSC))	PA, sedentary lifestyle, life satisfaction and intention to be physically active were assessed through validated questionnaires.	Having a low PA level, low cardiorespiratory fitness, low lower body muscular fitness and more screen time increase the risk of having low intention to be physically active in adolescents.	Increasing PA, PF and life satisfaction as well as reducing screen time could positively influence the intention of adolescents to be physically active.
**18**	F=50.3, M=49.7; *n*=578	Individual, environment, and family[k]	Accelerometry (ActiGraph devices)[j]	Study was designed to examine the social and environmental influences on unhealthy weight gain and activity in adolescents.	For boys, greater self-efficacy, and baseline MVPA remained significantly associated with MVPA at follow-up. For girls, baseline MVPA and barriers significantly predicted MVPA at follow-up in the full model.	Early PA habits are the most important predictor of PA levels in adolescence

**19.**	N/A; *n*=237	Family (Parent support)	Questionnaire (self-report PA)	Confirmatory factor analysis was used to estimate factor structure of the questionnaire.	Parents' assessments of the importance of PA and physical ability, which was considered as one of the factors related to children's PA.	Physically active parents evaluated the importance of PA of their children more than physically non-active parents.
**20.**	F=48.2, M=51.8; *n*=303	Individual (Demo and behavior), Family and peer support, home, and neighborhood environment	Questionnaire (self-report PA, Children's Leisure Activities Study Survey questionnaire-Chinese version (CLASS-C))	Hierarchical multivariable regression analyses were conducted to explain PA and SBBs.	Participation in school sport teams and self-efficacy was positively associated with PA in boys. Girls who reported participation in school sport teams, who perceived more peer support, had a more supportive home PA environment, and spent more time doing homework were more physically active. Family support for PA was negatively associated with boys' SBBs.	Sex differences existed among the individual, behavioral, social and environmental factors related to PA and SBBs.
**21.**	F=53.3-47.1, M=46.7-52.9; *n*=137593	GDP	Questionnaire (Health Behavior in School-Aged Children Study (HBSC))	Health Behavior in School-Aged Children Study (HBSC), which consisted of a questionnaire that was administered in the classroom setting.	PA interventions should be a fundamental component of health campaigns aimed at reducing the global obesity epidemic.	The likelihood of being overweight was significantly lower in a dose-response manner with higher PA participation in 29 out of the 33 countries examined (88%).
**22.**	F=48.9, M=51.1; *n*=3956	Individual, family, peer, school, environmental characteristics[l]	Questionnaire (self-report PA)	The present study utilized data from the Kindergarten (K) cohort of the Longitudinal Study of Australian Children (LSAC).	In the final model, the likelihood of participating in non- organized PA at age 13 was significantly predicted by sex, enjoyment of PA, number of siblings and home computer use at age 11. Females also tended to spend less time in non- organized PA at age 13, compared with males.	Significant predictors of decline in non-organized PA were socioeconomic position.
**23.**	F=46, M=54; *n*=535	Family (parental) and peer support	Questionnaire (Newly established survey, Self-reported data)	A survey was completed by adolescents at two single-sex high schools in Ohio.	Adolescents who received parental encouragement to exercise and who had an exercising friend engaged in significantly more days of PA.	Perceived benefits of PA differed significantly based on whether the respondent received parental encouragement and had a friend who exercised.
**24.**	F=55.1, M=44.9; *n*=1787	Individual (Demo and behavior), Family and peer support, home, and neighborhood environment	Questionnaire (self-report PA, Children's Leisure Activities Study Survey questionnaire-Chinese version (CLASS-C))	Hierarchical multiple logistic models were developed based on a conceptual framework of factors related to PA.	Girls were twice as likely to be inactive; Paternal education was inversely associated with inactivity. Adolescents living in neighborhoods without sidewalks were 1.3 times more likely to be inactive. Lack of extracurricular exercise and fewer sports meetings were associated with physical inactivity. Difficult access to community recreational facilities and concerns about safety in the neighborhood were associated with inactivity	Strategies to promote PA adolescents should integrate associated factors at household, school, and community levels and address the important gender differences in factors associated with physical inactivity in youth.

Supplemental Data A - Systematic Review and Meta-analysis Data

**25.**	F=57.9, M=42.1; *n*=2710	Family, Peers	Questionnaire (A short version of the International PA Questionnaire (IPAQ))	A structural equation modeling analysis was conducted to evaluate direct associations between social support from family and friends and the practice of PA in adolescents.	PA practiced by adolescents tend to be influenced by their social environments. Adolescents with a higher perceived social support from family presented higher levels of PA. Also, the high perceived social support from friends was directly related to higher levels of PA.	Sample size, and only individual models with social support from family or friends had adequate adjustment indexes, possibly indicating that social support and its effects on adolescents are inherent to social environments.
**26.**	F=54.1, M=45.9; *n*=660	Sitting time, individual[m], and peers (social support)	Questionnaire (Self-reported data, International PA Questionnaire)	Data were analyzed using a hierarchical linear model framework.	significant similarities between reciprocal best friend dyads for PA and sitting time, and for sitting time in non-reciprocal best friends. Best friend gender, regular sports practice of the person, perceived exercise barriers of the best friend and best friend social support were the best predictors for PA.	best friends have similar PA levels and some characteristics of the best friend seem to be factors that contribute to increased PA level.
**27.**	N/A; *n*=864	Individual and Family (individual level, home level, preschool level)	Accelerometer (Actigraph W-GT3X)[a,d]	Multilevel regression analyses were conducted in multiple steps; calculation of marginal and conditional R2 values occurred in the final phase	A set of correlates of preschool children's sedentary time and PA participation, revealed children's individual characteristics were associated with children's ST and PA.	Study introduced novel information about factors explaining the variance in preschool children's ST and PA.
**28.**	F=47, M=53; *n*=2018	Active transport to school (ATS)	Questionnaire (Self-reported data)	Multivariate binary logistic regression models were used to compare active transport to school correlates in students using active transport to school versus bus and car users.	ATS positively associated with PA	ATS can contribute one-quarter of total daily MVPA in adolescents.
**29.**	F=53.1, M=46.9; *n*=2779	Forty-six possible personal, social and environmental correlates of MVPA	Questionnaire (Self-reported PA)	Confirmatory (CFA) and exploratory factor analyses (EFA) were used to determine the factor structure.	The mix of personal and social correlates showed the strongest association with MVPA	Using factor analysis to show that correlates of MVPA may load onto the same factor across levels of the social ecological model
**30.**	F=55.1, M=54.9; *n*=1699	Individual, Social Environment, Physical or Built Environment	children's independent mobility (CIM)	Gender-stratified linear mixed-effects models while controlling for site, area-level SES, and type of urbanization.	Individual correlates including child grade, language spoken at home, car ownership, and phone ownership were associated with CIM and associated to PA.	children's independent mobility (CIM) could be one such determinant on PA, this article provide some insights of it.

**31.**	N/A; *n*=1369	Family (Parental PA habits, TV viewing, PC use, and barriers)	Questionnaire (self-report PA)	A principal component analysis (PCA) identified child and parental patterns, which were tested for association with child participation in extracurricular sports (ES).	SES, weight, parent's PA, and parental reported barriers were significantly associated with frequency of participation in ES.	the holistic approach of the present study successfully identified modifiable risk behaviors, which is of value when planning future PA interventions.
**32.**	F=49.2, M=50.8; *n*=834	Family (parental PA and type of parental PA)	Questionnaire (self-report PA)	Multinomial logistic regression was applied to assess associations between parental and child physical behaviors.	Both parents active was significantly associated with frequent participation in more sports both in girls and boys but a strong relation according to gender was found.	Family-based coactivity interventions during child development and reinforce children's PA.
**33.**	F=54.5 M=55.5; *n*=1223	Peers(friendships)	Accelerometer (ActiGraph wGT3X-BT)[a,d]	Children named up to four school friends and same-sex school-based friendship networks were constructed.	Both MVPA and sedentary time were found to be associated via the friendship networks, for both boys and girls.	PA and ST of children are associated with the PA and ST of others within the same-sex friendship network.
**34.**	F=48.5, M=51.5; *n*=2126	Family (Social Class, Parental Values, Cultural Capital)	Questionnaire (self-report PA)	A Principal Components Analysis and confirmatory factor analysis, binary logistic regression explored three major factors.	Cultural capital is the strongest, most consistent predictor of activity participation in our analysis; PA spectrum is greatly gendered under Asia cultural context. After-school activity also functions as a means of gender socialization, which might also set up a roadblock for non-traditional children.	children's after-school activity participation not only is quantitatively class-divided, but also qualitatively differs.
**35.**	F=57.4, M=52.6; *n*=1896	Peer (friendship network)	Questionnaire (Self-reported data)	The SAB model simultaneously estimates changes in the network and changes in individual BMI and PA.	Friendships were more likely among adolescents who engaged in greater PA and who were similar to one another in BMI and PA.	Adolescents' school-based friends might influence their BMI and PA and that adolescents' health shapes their friendships
**36.**	F=49.9, M=50.1; *n*=2660	Individual (Demo and behavior), Family and peer support, home and neighborhood environment	Questionnaire (The PA Questionnaire for Older Children (PAQ-C))	Potential risk factors for physical inactivity were identified with hierarchical multivariate logistic regression.	Schools having one to two sport meetings per year, availability of a play yard, older age group, and male gender, household, school, and community factors were related to PA.	The accessibility to public recreation facilities and the school location were significantly associated with physical inactivity in girls.

**37.**	F=51, M=49; *n*=138014	Physical, socio-cultural, economic, political factors	Questionnaire (self-report PA, Health Behavior in School-Aged Children Study (HBSC)	Multilevel regression models examined the influence of four types of country-level environmental factors on individual-level MVPA and VPA.	Lower temperature, higher community safety, lower average national household income and a weaker physical education policy were significantly associated with more MVPA. Greater urbanization, lower temperature, higher adult PA and higher average national household income were significantly associated with more VPA.	National differences in the physical, socio-cultural and economic environment were related to adolescent PA.
**38.**	F=48.3, M=51.7; *n*=288	Individual (perceived competence, enjoyment), school social and physical environments	Questionnaire (Self-reported data)	Analysis of variance, bivariate correlations among the study variables, and hierarchical multiple regression analyses were completed.	Perceived competence and enjoyment predicted PA for boys, while perceived competence was the only predictor for girls.	The results support associations between predisposing factors and self-reported PA as theorized within the social ecological model and the YPAP model.
**39.**	F=46.6, M=53.7; *n*=680	School PA promotion	Accelerometers (ActiGraph GT3X-Plus)[n]	The participants were randomly assigned to one of four treatment conditions. Differences in the outcome measures and characteristics of the study sample were examined using univariate F-test for continuous variables or contingency table (chi-square test or Cramer’s V) for categorical variables.	School PA promotion program significantly increase student's weekday and weekend MVPA.	School PA promotion program significantly increase student's weekday and weekend MVPA.

Supplemental Data A - Systematic Review and Meta-analysis Data

Note. [a]ActiGraph GT3×+ (Actigraph LLC, Pensacola, FL), [d]for seven consecutive days, Physical Activity Questionnaire for Adolescents.

[b] MD = mother-daughter, MS = mother- son, FD = father-daughter, and FS = father-son dyads. 357 MD, 393 MS, 117 FD, 133 FS

[C]IG = Intervention group, CG = Control Group; CG (F=263, M=222); IG (F=129, M=86)

[e]GDP = Gross domestic product, GDP per capita was discussed in this study

[f]Actigraph LLC, Shalimar, FL

[g]Yamax Digiwalker SW-701 (Optimal Health Products and Services, San Antonio, TX)

[h]multiday memory (MDM) pedometers (Model NL- 2000; New Lifestyles, Inc, Lees Summit, MO)

[i]Actiheart devices (Actiheart software version 13.1.4., CamNtech, Cambridge, UK), accelerometry and heart rate monitoring for up to six consecutive days including two weekend days (epoch length 15 s, continuous 24 h recording)

[j]ActiGraph model 7164 (ActiGraph, LLC, Pensacola, FL) was used to collect 7 days of PA data using 30-s epochs (data collection intervals).

SBBs = screen-based behaviors; ST = Sedentary time

[k]Pubertal status, self-efficacy, PA enjoyment, perceived barriers, height, weight and percent body fat (PBF), screen time, sports team participation, perceived parent support, perceived peer support, home PA environment, Perceived neighborhood safety, ease of mobility, and walkability

[l]demographic/physical characteristics (e.g., sex, body mass index), psychosocial characteristics (e.g., temperament, mental health), behavioural characteristics (e.g., sport participation, screen time), family characteristics (e.g., number of siblings, parental PA), school characteristics (e.g., type of school), peer relations (e.g., bullying) and neighbourhood characteristics (e.g., rurality).

[m]perceived exercise benefits and barriers, physical self-perception

[n]ActiGraph GT3X-Plus accelerometers (ActiGraph, Pensacola, FL, USA)

[o]origenal reported as 57.76 (8.73) months;

1 = Aaltonen et al. (2018); 2 = Appelhans & Li (2016); 3 = Beets et al. (2006); 4 = Bélanger-Gravel et al. (2015); 5 = Bergh et al. (2012); 6 = Best et al. (2017); 7 = Brug et al. (2012); 8 = Cairney et al. (2015); 9 = Davison & Jago (2009); 10 = Davison, Cutting, & Birch (2003); 11 = Duncan et al. (2007); 12 = Duncan et al. (2008); 13 = Duncan, Duncan, & Strycker (2005); 14 = Eichinger, Schneider, & De Bock (2017); 15 = Fu et al. (2020); 16 = Garcia et al. (2016); 17 = Grao-Cruces et al. (2015); 18 = Hearst et al. (2012); 19 = Hein (2015); 20 = Huang, Wong, & Salmon (2013); 21 = Janssen et al. (2005); 22 = Kemp et al. (2021); 23 = King, Tergerson, & Wilson (2008); 24 = Li et al. (2006); 25 = Lisboa et al. (2021); 26 = Lopes, Gabbard, & Rodrigues (2016); 27 = Määttä et al. (2020); 28 = Mandic et al. (2015); 29 = Miller (2019); 30 = Riazi et al. (2019); 31 = Rodrigues, Padez, & Machado-Rodrigues (2018a); 32 = Rodrigues, Padez, & Machado-Rodrigues (2018b); 33 = Salway et al. (2018); 34 = Shih & Yi (2014); 35 = Simpkins et al. (2012); 36 = Trang et al. (2009); 37 = Weinberg et al. (2019); 38 = Zhang, Thomas, & Weiller (2015); 39 = Zhou et al. (2019)
