# Supplementary material for: Correlations between Physical Activity Participation and the Environment in Children and Adolescents: A Systematic Review and Meta-Analysis Using Ecological Frameworks
Source: Int J Environ Res Public Health. 2021 Aug 28;18(17):9080. doi: 10.3390/ijerph18179080 (PMC8430662; doi:10.3390/ijerph18179080)
Supplement: Supplementary file 1 [file ijerph-18-09080-s001.zip › Supplemental File B.pdf]

Supplemental Data B

Visualization matrix

The co-occurrences map was generated based on the algorithm which used a similarity measure known as the association strength (Van Eck & Waltman, 2010). This similarity measure was also referred to as the proximity index (PI) or as the probabilistic affinity index (AI). According to the bibliography association strength, the similarity s_{ij} between two items i and j is calculated as:

$$s_{ij} = \frac{c_{ij}}{w_i w_j}$$

Then, "where c_{ij} denotes the number of co-occurrences of items i and j and where w_i and w_j denote either the total number of occurrences of items i and j or the total number of co-occurrences of these items; It can be shown that the similarity between items i and j calculated using the above algorithm is proportional to the ratio between on the one hand the observed number of co-occurrences of items i and j and on the other hand the expected number of co-occurrences of items i and j under the assumption that occurrences of items i and j are statistically independent "(Van Eck & Waltman, 2010, p. 531). The researcher referred to Van Eck and Waltman (2010) for an extensive discussion of the advantages of the association strength over other similarity measures, such as the cosine and the Jaccard index.
