# Supplementary material for: Correlations between Physical Activity Participation and the Environment in Children and Adolescents: A Systematic Review and Meta-Analysis Using Ecological Frameworks
Source: Int J Environ Res Public Health. 2021 Aug 28;18(17):9080. doi: 10.3390/ijerph18179080 (PMC8430662; doi:10.3390/ijerph18179080)
Supplement: Supplementary file 1 [file ijerph-18-09080-s001.zip › Supplemental File C.pdf]

**Supplemental Data C**

*Europe countries in selected studies*

Europe included 7, 29, and 34 countries in three studies (Brug et al. (2012); Weinberg et al. (2019); Janssen et al. (2005)) respectively.

Seven countries included (Belgium, Greece, Hungary, Netherlands, Norway, Slovenia, and Spain).

Twenty-nine countries included (Austria, Belgium Flemish, Belgium French, Canada, Croatia, Czech Republic, Denmark, England, Estonia, Finland, France, Germany, Greece, Greenland, Hungary, Ireland, Israel, Italy, Latvia, Lithuania, Macedonia, Malta, Netherlands, Norway, Poland, Portugal, Russia, Scotland, Slovenia, Spain, Sweden, Switzerland, Ukraine, United States, and Wales).

Thirty-four countries included (Austria, Belgium Flemish, Belgium French, Bulgaria, Croatia, Czech Republic, Denmark, England, Estonia, Germany Greece, Hungary Ireland, Italy, Latvia Lithuania, Netherlands, Norway, Poland, Portugal, Romania, Scotland Slovakia, Slovenia, Spain, Sweden, and Wales).
